# Quality of life of individuals submitted to vestibular rehabilitation

**DOI:** 10.1016/S1808-8694(15)30657-1

**Published:** 2015-10-19

**Authors:** Olívia Helena Gomes Patatas, Cristina Freitas Ganança, Fernando Freitas Ganança

**Affiliations:** 1 Specialized in Human Communications Disorders. MSc Student - Human Communications Disorders Graduate Program - UNIFESP; 2 PhD in Sciences - Human Communications Disorders Graduate Program - UNIFESP -EPM, Adjunct Substitute Professor - Hearing Disorders Course - Speech and Hearing Therapy Program - UNIFESP-EPM; 3 PhD in Otorhinolaryngology. Adjunct Professor, Head of the Vestibular Rehabilitation Program - Neurotology Course - UNIFESP-EPM. Professor - MSc Graduate Program - Social Inclusion and Vestibular Rehabilitation Program - UNIBAN. Universidade Federal de São Paulo - Escola Paulista de Medicina

**Keywords:** quality of life, rehabilitation, dizziness, vertigo

## Abstract

Balance disorders affect social, family and professional activities. Vestibular rehabilitation can reduce the impact of these disorders on the quality of life of individuals with vertigo.

**Aim:**

to study the influence of vestibular rehabilitation on the quality of life of individuals, correlating it with gender, age, results from computerized vectoelectronystagmography and vertigo. Study type: Retrospective.

**Materials and Methods:**

Twenty-two individuals were submitted to customized vestibular rehabilitation and the Brazilian Dizziness Handicap Inventory - DHI before and after vestibular rehabilitation. Results from this questionnaire were correlated with gender, age, vestibular assessment and the presence of vertigo.

**Results:**

all the DHI scores reduced significantly after vestibular rehabilitation. There were no differences among genders; adults and elderly patients; irritative peripheral vestibular syndromes; deficiency syndromes and normal exams; the presence or absence of vertigo.

**Conclusion:**

all the individuals had improvements in their quality of life after customized vestibular rehabilitation.

## INTRODUCTION

Body balance, the capacity to keep oneself upright or to perform body movements without oscillation or falls is fundamental in order to adopt and keep postures, and it also allows for harmonious movements, physical and mental comfort. Disorders that affect this capacity can cause important clinical manifestations such as unbalance, gait deviations, instability, feeling of floating, falls, and others, and vertigo is the most common complaint.^1^,^2^

Numerous authors reported that the intensity, duration and prevalence of the clinical manifestations that follow vestibular disorders frequently affect family life, social and professional activities, bringing about physical, economical and psychological losses such as loss of self-confidence, depression and frustration, and also cause a reduction in concentration and performance, ultimately causing a worsening in Quality of Life.^1^,^3^, ^4^, ^5^

Aiming at assessing the Quality of Life (QL) or a patient's functional capacity, numerous tools have been proposed and used. Among them, we must stress the Dizziness Handicap Inventory (DHI), created and validated by Jacobson and Newman^6^, which assesses the patient's self perception of the incapacitating effects caused by dizziness. Such tool has been translated and culturally adapted by Castro et al.^7^ - the Brazilian DHI.

It is believed that the QL assessment can be used in daily practice to measure the contribution of clinical treatment in reducing the impact of chronic diseases in the daily lives of the patients.^8^,^9^

One of the currently used treatment for vertiginous patients is Vestibular Rehabilitation (VR), which aims at reducing dizziness and body instability and has been an important and effective strategy used to treat patients with body balance disorders, improving competence and well being in the performance of daily activities, providing a significant improvement in their quality of life.^1^,^3^,^4^,^9^, ^10^, ^11^, ^12^, ^13^, ^14^

It is believed that VR success may be influenced by some factors such as patient's age, compliance as to the practice of physical exercises, emotional state, medication use and the presence of central nervous system diseases which may compromise the structures associated with the neuroplasticity of the vestibular system.^4^

It is important to analyze the results from the VR programs. One efficient way to measure the success of chronic disease interventions would be the systematic use of QL assessment tools, such as the DHI, before, during and after the treatment period. The literature reports that the DHI could be a good tool to assess follow up and reevaluate the success of the rehabilitation program. Nonetheless, there are very few studies about the quality of life of individuals with vestibular disorders.

Knowing the importance of VR in the neurotological treatment proven in the pertaining literature and the large number of patients who complain of dizziness and other associated symptoms, this paper aims at checking the influence of VR on the Quality of Life of individuals, correlating the variables analyzed in the DHI with aspects such as gender, age, conclusion regarding the computerized vector-electronystagmography test and the presence or absence of vertigo.

## MATERIALS AND METHODS

This project was analyzed and approved by the Ethics in Research Committee under protocol # 0304/05.

We analyzed the medical charts of 22 patients seen at the Neurotology Ward who had been submitted to vestibular rehabilitation from 2002 to 2005.

The sample was made up of 13 men (59%), aged between 16 and 87 years, with mean age of 59.2 years; and nine women (41%), with ages between 36 and 77 years, mean age of 56.3 years.

All the patients were submitted to an interview, ENT evaluation, and audiological assessment with tonal and vocal audiometry, impedance measures and vestibular test by means of a digital vector-nystagmography prior to starting vestibular rehabilitation (VR). The patients complained of dizziness and had a diagnostic hypothesis of chronic peripheral vestibular syndrome (dizziness for more than 3 months).

These individuals answered the Brazilian DHI7 - Dizziness Handicap Inventory - at the beginning and at the end of the treatment process. This questionnaire has twenty five questions with the following answer options: “yes”, “no” or “sometimes”. Four points were given to “yes” answers, zero was given to “no” answers. Two points were given to each answer of “sometimes”. The maximum score was of one hundred points, and the higher the score, the greater was the impact of dizziness in the patient's QL. Three aspects were separately analyzed, and the sum of these scores at the end gives us the final score. Thus, there are seven questions assessing physical aspects, nine assessing emotional issues and 9 evaluating functional issues (Annex I). These aspects have an important role to play in the quality of life of dizzy patients.^7^,^8^

VR was made up of customized exercises according to patient complaint, clinical aspects and vestibulometry findings. The exercises proposed initially could be modified according to the clinical evolution of the patient during treatment, considering the improvement or not of vestibular symptoms. The exercises used were primarily taken from protocols established by Cawthorne^15^, Cooksey^16^, Herdman - Exercises to enhance stabilization in static and dynamic posture, to enhance vestibular adaptation and alternative strategies and strategies to enhance gaze stabilization^17^; Exercises from Davis and O'Leary^18^; Exercises from the Associazione Otologi Ospedalieri^19^.

ANNEX I **Disability caused by dizziness questionnaire / Brazilian DHI**

Patient's ID

Name:___________________________________Age (years):**_____** Birth Date:**_____**/**_____**/**_____**

Address:_________________________________________________________________________

Telephone #s:____________________HD:____________________________________________

**Table:** 

DHI			ANSWERS	
		YES	SOMETIMES	NO
ASPECT	QUESTIONS		SCORE	
		(4)	(2)	(0)

Physical1. Does looking up worsens your dizziness?

Emotional2. Do you feel frustrated because of your disease?

Functional3. Do you refrain from work or leisure travels because of you condition?

Physical4. Do you feel clinically worse when walking along the corridors of a supermarket?

Functional5. Because of your problem, do you have any difficulties to lie in bed or get up from it?

Functional6. Does your disorder impair your participation in social activities such as going out for dinner, going to the movies, going out dancing or going to parties?

Functional7. Because of your disorder, do you have trouble reading?

Physical8. Do sport activities or home cleaning worsen your clinical condition?

Emotional9. Because of your disorder, do you feel afraid of leaving home alone?

Emotional10. Because of your disorder, do you feel uncomfortable (embarrassed) in the presence of other people?

Physical11. Do fast head movements worsen your clinical condition?

Functional12. Because of your disease, do you avoid heights?

Physical13. Does changing positions when lying in bed worsens your clinical condition?

Functional14. Because of your disorder, is it difficult to perform more vigorous domestic activities?

Emotional15. Because of your disorder, are you afraid that other people might think you are drunk?

Functional16. Because of you disease, is it difficult for you to walk alone?

Physical17. Does walking in the boardwalk worsen your clinical condition?

Emotional18. Do you have concentration impairment because of your condition?

Functional19. Because of your disease, do you have difficulties to walk in the dark?

Emotional20. Because of your condition, are you afraid of being home alone?

Emotional21. Because of you disease, do you feel impaired?

Emotional22. Because of your disorder, have you had relationship problems with friends or family?

Emotional23. Because of your disorder, do you feel depressed?

Functional24. Does you disorder interfere in your professional activities?

Physical25. Does bending over worsens your clinical condition?

SCORE

Physical subscale: ___________ points Functional subscale: ________ points

Emotional subscale: _______ points TOTAL: ________ points

Rehab was given once a week, by a trained hearing therapist, and the patients were educated in order to do the exercises at home, twice or thrice a day, for at least six weeks. Nonetheless, depending on the patient's progress or on factors not directly associated with treatment, the patients occasionally had a higher or lower number of sessions.

We calculated the mean mathematical value of the scores obtained by the patients in the Brazilian DHI, regarding physical, emotional and functional aspects; and as to the total score before VR, minus the mean value of the scores obtained after the VR, in order to obtain a value indicative of the degree of improvement perceived by the patient. Thus, the higher this pre and post VR difference, the greater is the improvement in quality of life provided by the VR. This pre and post VR difference was analyzed in relation to the following variables: gender, age, results of the computerized vector-electronystagmography and the presence of vertigo.

For this study we used the following non-parametric tests: Wilcoxon, Mann-Whitney, Kruskal-Wallis, Spearman correlation and the Correlation Test. In complementing the descriptive analysis, we used the technique of confidence interval for the average. Statistically significant comparison levels (p<0.05) were marked with asterisks in the charts.

## RESULTS

All the patients had associations between different types of non-rotational dizziness, and some also had vertigo (rotational-type of dizziness).

The non-rotational-types of dizziness the individuals had more often were: a feeling of unbalance (n = 8; 36.36%), instability (n = 8; 36.36%), feeling of falling (n = 4; 18.18%), dizziness without other specifications (n = 4; 18.18%), of light-headedness or heavy head (n = 3; 13.63%), oscilopsia (n = 3; 13.63%), kinetosis (n = 3; 13.63%), floating (n = 2; 9.09%) and gait deviation (n =2; 9.09%). Of these individuals, eight (36.36%) also complained of vertigo (rotational-type of dizziness).

Other associated symptoms which appeared more frequently were: tinnitus (n = 13; 59.09%), neurovegetative manifestations (n = 8; 36.36%) and headache (n = 5; 22.72%).

Of the 22 individuals sampled, three (13.63%) had normal results seen at the vestibular test; 11 (50%) had Irritative Peripheral Vestibular Syndrome (6 unilateral and 5 were bilateral); 8 (36.36%) with Deficitary Peripheral Vestibular Syndrome (3 unilateral and 5 bilateral).

In the treatment process we used at least 3 and a maximum of 9 protocols, with an average of 5.9 different protocols used for each patient, as a basis for a tailor-made vestibular rehabilitation program. The most used protocols were: Herdman's (100%), Cawthorned's and Cooksey's (95.45%) Exercises to Enhance Static and Dynamic Posture Stabilization (100%); Herdman's Exercises to Enhance Vestibular Adaptation (95.45%); Exercises from the Associazione Otologi Ospedalieri Italiani (95.45%).

In the treatment process, we used an average of 15 exercises per individual, varying between a minimum of 8 and a maximum of 24 different exercises. The most used exercises were: move the head sideways fixing the eyesight on a specific point on the wall or on a card (n=19; 86.36%); move the head up and down fixing the eyesight to a specific point on the wall or on a card (n=19; 86.36%); walk and alternate gaze to the right and left (n=17; 77.27%); to march in the same spot on an irregular surface (n=14; 63.63%); walk flexing and extending the head (n=11; 50%).

In average, the individuals underwent 9 vestibular rehabilitation (VR) sessions, varying between a minimum of 6 and a maximum of 22 sessions.

Before being submitted to tailor-made VR, the individuals had, in average, the values of 17.09; 17.36; 17.82 and 52.27 in the physical, emotional, functional aspects and in the total score of the Brazilian DHI, respectively.

After VR sessions, the individuals had mean values of 8.45, 9.73, 9.36 and 27.45 in the physical, emotional and functional aspects and in the total score, respectively.

When we analyze the sample as a whole, we see that patients had better DHI scores in all the aspects assessed in the questionnaire. Graph 1 compares the pre and post vestibular rehab values of the total sample. We found statistically significant differences between the pre and post Vestibular Rehab considering all aspects associated with the Brazilian DHI.

**Graphic 1:**
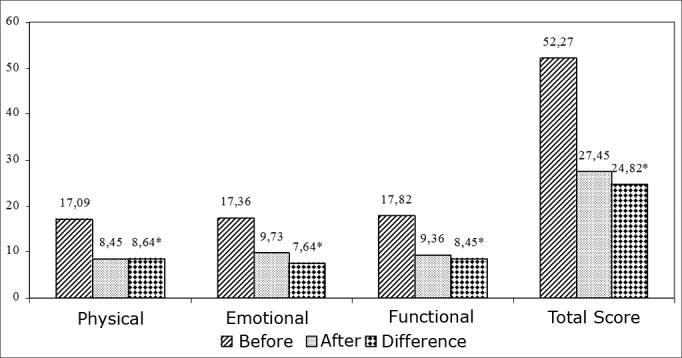
Comparing pre and post vestibular rehabilitation scores of the total sample.

Graph 2 shows the reduction in the total score and in the different aspects, obtained after VR, according to gender. We observed that there was no difference between genders as to the improvement in the Brazilian DHI scores in any of the aspects encompassed in the questionnaire - physical, emotional and functional.

**Graphic 2:**
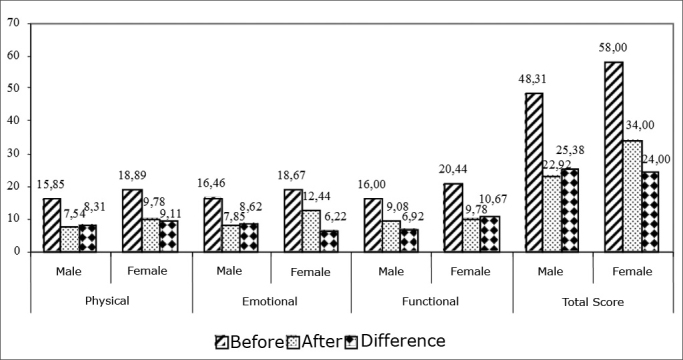
Comparing genders as to the Brazilian DHI difference before and after VR.

In the sampled individuals, 11 were 60 years old or less and 11 individuals were older than 60 years. We noticed that although there is a difference in the reduction values between these two groups, they were not statistically significant. Nonetheless, the improvement in the Brazilian DHI after VR was statistically significant in both groups - adults and elderly. Graph 3 shows the comparison of adults and elderly patients as to the reduction in the Brazilian DHI scores.

**Graphic 3:**
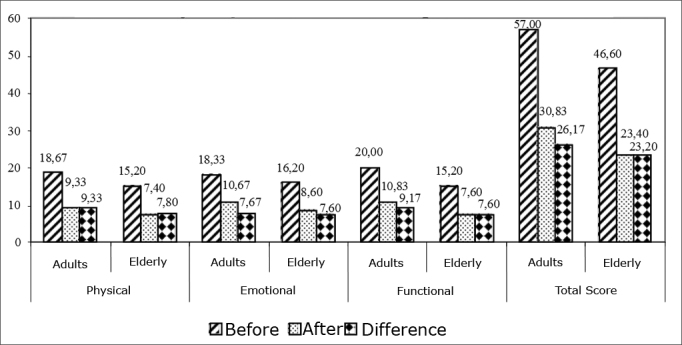
Comparing adults and the elderly as to the differences in the Brazilian DHI after VR.

As far as the vestibular assessment is concerned, we observed that in the final/total score, those patients with deficitary peripheral vestibular syndrome (DPVS) had better improvement levels after VR, according to results from the Brazilian DHI, when compared to those patients with irritative peripheral vestibular syndrome (IPVS), and the later more than the patients with normal tests. This same descending order of improvement after VR was seen when we specifically analyzed physical and functional aspects. Notwithstanding, these differences were not statistically significant. The comparison of the Brazilian DHI with the results from the vestibulometry is shown on Graph 4. The comparison of the values obtained from the Brazilian DHI by individuals who had only non-rotational dizziness and those who had association between non-rotational dizziness and vertigo is shown on Graph 5. Although there are differences between the patients who had only non-rotational-type of dizziness and those that besides this one also had vertigo as to the reduction of the Brazilian DHI score, the differences were not statistically significant. Even then, in both groups the total score after VR was significantly lower when compared to those before treatment.

**Graphic 4:**
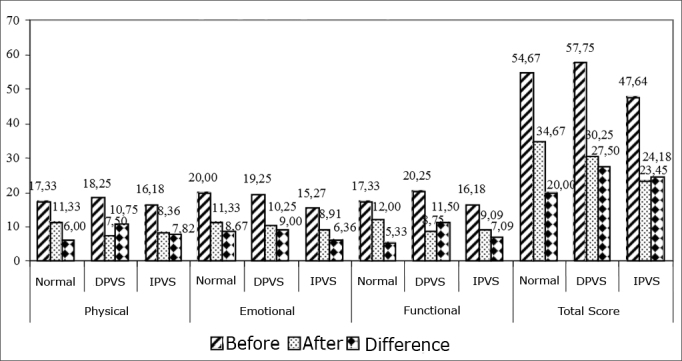
Comparing the conclusions found at vector-electronystagmography as to the differences in the Brazilian DHI.

**Graphic 5:**
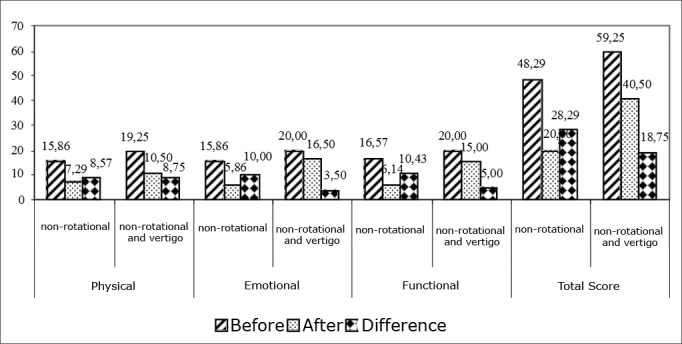
Comparing subjects with non-rotational dizziness and those with vertigo as to the differences in DHI.

## DISCUSSION

In the present investigation, we used tools such as the Dizziness Handicap Inventory - Brazilian DHI in order to compare quality of life before and after vestibular rehabilitation (VR).

The physical aspects assessment allows us to see the relationship between eyes, head and body movements and the onset or worsening of dizziness. The emotional aspect helps assess frustration, fear of leaving home alone or that of being home alone, shame of the disease clinical manifestations, concern with one's self-image, concentration difficulties, a feeling of incapacity, and depression problems of social and family relationship in dizzy patients. And finally, the functional aspect helps to find impairments in the performance of professional, domestic, social and leisure activities, and also to assess dependence to perform certain tasks, such as walk with help and difficulties to walk inside the house in the dark.^20^

It is believed that a reduction greater than or equal to 18 points resulting from the difference among DHI scores pre and post treatment may indicate benefits achieved with the vestibular rehabilitation technique employed6. Thus, the difference seen in the sample of the present study (pre and post difference = 24.82 points) reveals that the reduction in total values of the Brazilian DHI was significant, in other words, the individuals had an important improvement in their quality of life after VR.

In agreement with our study, Meli et al. assessed 43 patients with chronic dizziness submitted to outpatient VR and home exercises twice a day, and there was an 18.04 points reduction in the DHI, and each one of the three subscales also showed constant reductions after VR. There was no significant difference between genders and also regarding the age ranges studied (<45 and ⩾45 years) as to the drop in DHI scores. The authors concluded that the vestibular rehabilitation improves quality of life by reducing the challenge and improving the skills needed to perform daily activities.^14^

Our results are in partial agreement with those found by Badke et al.^13^. These authors assessed balance recovery and the disability caused by dizziness in 32 patients after a customized vestibular rehabilitation program. DHI scores improved moderately among these individuals; however this improvement was statistically significant only for the functional scale. The authors believe that the DHI result may have been influenced by a number of factors, including the level of compensation, symptoms acceptance and the degree of compliance with the exercises program.^13^

Our findings corroborate those from Cohen and Kimball, from 2003, who submitted 53 patients with chronic peripheral vestibular diseases to a VR program. They assessed the impact of dizziness on the quality of life using DHI before, during and after treatment. They also observed a reduction in the intensity and frequency of vertigo and a drop in DHI scores after VR, which continue to reduce throughout the six months that followed. The improvement seen by the authors was not affected by gender, age or time of vertigo onset. Independence to perform daily activities, the frequency and intensity of vertigo and psychosocial factors also improved significantly after rehabilitation.^21^

In 2005, Nishino et al. performed a study on the vestibular rehabilitation program in 37 patients with varied neurotological manifestations. VR was weekly performed in the outpatient ward, and at home on a daily basis, with specific exercise programs, considering the findings of the vestibular exam, clinical manifestations and, specially, the symptoms presented. The authors concluded that the customized VR program proved to be an effective treatment to reduce and eliminate symptoms, consequently improving the quality of life of patients with different clinical manifestations.^2^

The results obtained from the present investigation lead us to believe that all the individuals - adults and elderly; men and women - significantly benefited from vestibular rehabilitation in terms of quality of life, regardless of age and gender.

These results corroborate those from Cohen and Kimball^21^ and from Meli^14^ et al., in which they also observed that the improvement was not affected by gender, and they also showed that there was no significant difference in relation to age when DHI score reduction was considered.

In 2002, Whitney et al., assessed 23 patients with vestibular disorders, which were paired by gender, vestibular diagnosis and results from the vestibular function tests with 23 elderly individuals. All patients were submitted to a VR program with twice a month visits and exercises to be performed at home. One of the measures of clinical improvement used by the authors was defined as a change in the total DHI score of at least 18 points. After rehabilitation, there was a global improvement in both groups, without statistically significant improvement as to the DHI and as to the ratio of patients with clinical improvement. Age was not a significant factor used to predict VR results.^22^

In a similar fashion, Bittar et al., studied 35 elderly patients treated by VR and observed that 18 (51.4%) showed total improvement and 7 patients (20%) reported a partial symptoms improvement. We did not have a statistical difference between the group of elderly and the total group. The authors stated that the geriatric population responds to treatment just as well as the younger population, although most of the individuals in the former group require more follow up sessions in order to obtain the same result.^23^

Despite being a common concern among clinicians and patients, age is not necessarily associated with the loss of independence on daily life activities. These results showed that age is not necessarily associated with a reduced skill in recovering independence or improving vertigo after vestibular disorder.^21^

Although we did not see statistically significant differences in the Brazilian DHI scores between young adults and elderly patients, many studies showed that dizziness has a harmful effect on all the quality of life measures among elderly patients. Dizziness in elderly adults has been associated to a limitation in daily life activities, difficulty in walking, depression symptoms, cardiovascular disorders and sensorineural symptoms.^4^,^13^,^22^,^24^, ^25^, ^26^, ^27^

Alterations which pertain to aging in the systems associated to body balance, the greater likelihood of chronic-degenerative diseases, the chronic and often times use of multiple medications, among other factors seem to favor dizziness and worsen its intensity, causing physical, functional or emotional limitations in this age range.^24^, ^25^, ^26^, ^27^, ^28^

As far as the vestibular assessment is concerned. We observed that in the total score and physical and functional aspects, patients with deficitary peripheral vestibular syndrome (DPVS) had a more pronounced improvement after VR when compared to those patients with irritative peripheral vestibular syndrome (IPVS), and these more than those patients with normal tests. Nonetheless, there was no statistically significant difference in the Brazilian DHI score reduction among vector-electronystagmogram conclusions.

In a study involving 25 patients with dizziness and diagnostic suspicion of peripheral vestibular syndrome, Ganança et al.^28^ showed that patients with chronic dizziness had a worse quality of life in terms of physical, functional and emotional aspects of their lives seen on the Brazilian DHI. However, the authors stated that the DPVS are clinically correlated with vestibular disorders in which there is a total or partial reduction in vestibular function, usually having a worse prognosis when compared to the IPVS, thus they could be related to a worse quality of life^28^. Nonetheless, the authors did not aim at investigating vestibular post-rehabilitation development, which was our goal, and it was not possible to make a direct comparison.

In 2005, Segarra-Maegaki and Taguchi, assessed 12 patients by means of Vector-electronystagmography (VENG), and with results of Irritative Peripheral Vestibular Syndrome, who were submitted to the DHI. The differences in the pre and post treatment indexes were: for the physical scale, varied from zero to 18 points with an average of 7; for the functional scale from zero to 20 and mean value of 8.3; and for the emotional scale from zero to 28 and mean value of 5.5. The distribution of the total difference of DHI indices in the pre and post treatment varied from −4 to 68 with an average of 17.33. The authors obtained a change in diagnosis from Irritative Syndrome to Normal Vestibular Test in 75% of the cases after VR. And also, in that study there was a significant improvement in the quality of life of the patients. VR brought about benefits to the patients and DHI proved to be an efficient enough instrument to study VR benefits.^5^

All the individuals in this sample had some type of non-rotational dizziness, and some patients with complaints of associated vertigo. We noticed that the individuals who had only non-rotational dizziness had greater reduction indexes in all the Brazilian DHI scores and in the total score, despite the differences in the Brazilian DHI values not being statistically significant, when compared to those which had associations with vertigo.

Our findings corroborate those in the literature as it shows the importance of the Brazilian DHI as a tool to better quantify the improvement of patients submitted to vestibular rehabilitation. Based on the analysis provided by the Brazilian DHI, we can see that the aspect that was most changed by means of the vestibular rehabilitation was the functional one, which encompasses the losses in the performance of professional, domestic, social and leisure activities, and it also helps assess the patient's dependence in performing certain tasks, such as walking with help and difficulties to walk inside one's home in the dark, that is, vestibular rehabilitation improved the quality of life of the individuals as it provided them with benefits in the performance of the aforementioned activities.

## CONCLUSIONS

Individuals with peripheral vestibular syndrome submitted to vestibular rehabilitation had improvements in their quality of life. The improvement seen was not impacted by gender, age, results from the vestibular test or vertigo complaint.
